# Extracellular intersubunit interactions modulate epithelial Na^+^ channel gating

**DOI:** 10.1016/j.jbc.2023.102914

**Published:** 2023-01-14

**Authors:** Lei Zhang, Xueqi Wang, Jingxin Chen, Shaohu Sheng, Thomas R. Kleyman

**Affiliations:** 1 Departments of Medicine, University of Pittsburgh, Pittsburgh, Pennsylvania, USA; 2 Department of Nephrology, Hunan Key Laboratory of Kidney Disease and Blood Purification, The Second Xiangya Hospital, Central South University, Changsha, Hunan, China; 3 The Third Xiangya Hospital, Central South University, Changsha, Hunan, China; 4 Cell Biology, University of Pittsburgh, Pittsburgh, Pennsylvania, USA; 5 Pharmacology and Chemical Biology, University of Pittsburgh, Pittsburgh, Pennsylvania, USA

**Keywords:** Epithelial sodium channel, Protein–protein interaction, Protein crosslinking, Disulfide, Hydrogen peroxide, Protein domain, Allosteric regulation, Channel gating, Extracellular domain, Voltage clamp, Amiloride, ASIC1, acid-sensing ion channel 1, ENaC, epithelial Na^+^ channel, H_2_O_2_, hydrogen peroxide, Ipeak, peak current, Iss, steady-state current, MTSES, sodium (2-sulfonatoethyl) methanethiosulfonate, MTSET, [2-(trimethylammonium) ethyl] methanethiosulfonate bromide

## Abstract

Epithelial Na^+^ channels (ENaCs) and related channels have large extracellular domains where specific factors interact and induce conformational changes, leading to altered channel activity. However, extracellular structural transitions associated with changes in ENaC activity are not well defined. Using crosslinking and two-electrode voltage clamp in *Xenopus* oocytes, we identified several pairs of functional intersubunit contacts where mouse ENaC activity was modulated by inducing or breaking a disulfide bond between introduced Cys residues. Specifically, crosslinking E499C in the β-subunit palm domain and N510C in the α-subunit palm domain activated ENaC, whereas crosslinking βE499C with αQ441C in the α-subunit thumb domain inhibited ENaC. We determined that bridging βE499C to αN510C or αQ441C altered the Na^+^ self-inhibition response *via* distinct mechanisms. Similar to bridging βE499C and αQ441C, we found that crosslinking palm domain αE557C with thumb domain γQ398C strongly inhibited ENaC activity. In conclusion, we propose that certain residues at specific subunit interfaces form microswitches that convey a conformational wave during ENaC gating and its regulation.

The epithelial Na^+^ channel (ENaC) is a Na^+^ selective and non–voltage-gated channel blocked by low concentrations of amiloride. ENaC expression has been detected on the apical membrane of epithelial cells in organs including kidney, lung, colon, sweat and salivary glands, lingulae, and the reproductive tract. ENaCs are also expressed in nonepithelial cells, including endothelia, vascular smooth muscle, and dendritic cells (1, 2, 3). In the aldosterone-sensitive distal nephron, ENaC functions together with basolateral Na^+^/K^+^-ATPase, apical renal outer medullary potassium channel and sodium chloride cotransporter, regulates Na^+^ homeostasis and extracellular fluid volume, K^+^ homeostasis, and blood pressure (1, 2, 3, 4, 5, 6, 7, 8, 9, 10, 11).

ENaCs are members of a family of ion channels that are formed by subunits that share common structural features, including large well-organized extracellular domains. In the case of ENaC, heterotrimeric channels are formed by α (or δ), β, and γ subunits. Resolved structures of extracellular domains of ENaC and a related acid-sensing ion channel 1 (ASIC1) have been described as an outstretched hand holding a ball, with discrete regions formed by β strands or α helices, including the finger, thumb, knuckle, palm, and β ball domain (12, 13). Factors in the extracellular environment, including specific ions, peptides, and proteins, interact at sites within ENaC and related family members and modulate channel gating. In the case of ENaC, channel activity is regulated by extracellular Na^+^, Cl^−^, protons, metals, proteases, and sheer stress (3, 14, 15, 16, 17, 18, 19, 20, 21, 22, 23, 24, 25, 26, 27, 28). For example, Na^+^ interacts at a site within the extracellular region of the α subunit of ENaC, resulting in conformation changes that eventually reduce channel open probability, a phenomenon referred to as Na^+^ self-inhibition (3, 15, 29). However, the mechanistic details how these extracellular regulators impact ENaC gating remain elusive.

A five-residue track connecting the palm domain β11 and β12 strands, referred to as the β11–β12 linker, has been shown to have a role in ASIC1 desensitization (30, 31, 32, 33, 34, 35, 36). Specifically, structural studies suggest that a hydrophobic residue (L414) and an adjacent hydrophilic residue (N415) in this linker positions reorient toward the central vestibule in desensitized channels, compared with closed channels. This structural reorientation has been proposed to function as a molecular clutch, decoupling upper and lower parts of the extracellular domain, and facilitating a transition to the desensitized state (32). These structural transitions within the ASIC1 β11–β12 linker raised the possibility that this linker within ENaC subunits may also undergo a conformational change in association with gating transitions. Indeed, we previously suggested that L511 in human γ ENaC, homologous to L414 in ASIC1, interacts with an adjacent subunit through hydrophobic contacts. We speculated that the gain-of-function γL511Q mutation disrupted the hydrophobic interaction by introducing a hydrophilic side chain, facilitating a separation of the palm domain of γ subunit and the thumb domain of an adjacent subunit and stabilizing the channel in an open state (37). In support of this hypothesis, a recent ASIC1a study demonstrated that a hydrophobic patch stabilized L414 side chain when the channel is in a closed state and where structural transitions were deemed necessary for channel desensitization (34).

The five-residue track connecting the palm domain β11–β12 strands is conserved among ENaC subunits (Fig. 1*D*). A sole hydrophobic residue (Leu or Phe) is flanked by four hydrophilic residues including a negatively charged residue preceding human γL511 (mouse γL517). Mutations of this acidic residue in the α or γ subunit alter ENaC activity, with changes in either the Na^+^ self-inhibition response or the response to extracellular Zn^2+^ (38, 39, 40). We hypothesized that acidic residues in β11–β12 interact with polar residues of an adjacent subunit at the subunit interface and that these intersubunit interactions have important roles in the regulation of ENaC gating. To test this hypothesis, we engineered Cys substitutions in the β11–β12 linker and at sites in adjacent subunits that are predicted to be in close proximity. Channels were expressed in *Xenopus* oocytes that were treated with either oxidizing agents to induce disulfide bridges or reducing agents to break these bridges. We identified several pairs of introduced Cys residues where ENaC currents were altered in response to a reducing or oxidizing reagent.

**Figure 1:**
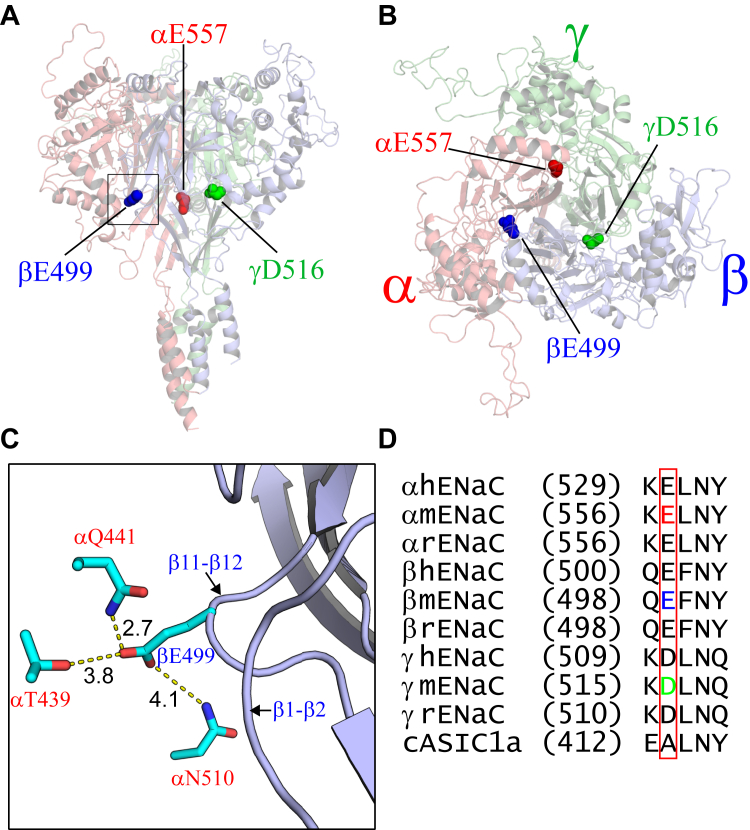
**Acidic residues of the β11–β12 linkers reside at subunit interfaces.***A* and *B*, locations of the acidic residues of the β11–β12 linkers in the mouse ENaC structural model. The trimeric model was previously built (41) and displayed by PyMol 2.4 (78). α, β, and γ subunits are showed in *red*, *blue*, and *green*, respectively. Side chains of αE557, βE499, and γD516 are presented as *spheres*. *A*, side view. *B*, top view. *C*, βE499 and its adjacent residues in the α subunit. The displayed area corresponds to the square in *A*. Side chains of the labeled residues are presented as *sticks* with carbon in *cyan*, oxygen in *red*, and nitrogen in *blue*. Distances between two residues were measured as the minimal distance between nonhydrogen atoms of the side chains of the two residues using PyMol. The three α subunit residues are the closest polar residues to βE499. *D*, sequence alignments of the α, β, and γ subunits of human (h), mouse (m), rat (r) ENaC, and chicken (c) ASIC1a. Numbers in the parenthesis represent the first residues of the amino acid sequence. Acidic residues of interest within the β11–β12 linker are framed in *red rectangle*. Among them, αE557, βE499, and γD516 of mouse ENaC are shown in *red*, *blue*, and *green*. ASIC1a, acid-sensing ion channel 1; ENaC, epithelial Na^+^ channel.

## Results

### Mouse ENaC model and its α/β subunit interface

Based on current knowledge of ENaC and ASIC structure–function relationships, we hypothesized that specific extracellular interdomain interactions involving β11–β12 linkers and adjacent sites located at a subunit interface have important roles in modulating ENaC gating. To test this hypothesis, we introduced Cys residues at specific sites within ENaC subunits and exposed expressed channels to either oxidizing agents to induce disulfide bridges or reducing agents to break disulfide bonds. To identify targets for engineering disulfide bridges, we utilized a mouse ENaC homology model recently built from the template of trimetric human ENaC structure (41) (Fig. 1, *A* and *B*).

At the α/β subunit interface in the mouse ENaC model, we identified three α-subunit polar residues in close proximity (less than 4.5 Å of minimal distance between side-chain oxygen or nitrogen atoms) to βE499 in the β11–β12 linker, including αN510 in β10 strand of the palm domain, αQ441 in α4 helix, and αT439 immediately preceding α4 helix of the thumb domain (Fig. 1*C*). These residues (βE499, αN510, αT439, and αQ441) were individually mutated to Cys, and wildtype or mutant channels bearing one or two Cys mutants were expressed in *Xenopus* oocytes. The functional effects of inducing or disrupting Cys crossbridges were assessed by two-electrode voltage clamp. Oocytes were treated with an oxidizing agent (H_2_O_2_) to induce disulfide bond formation, or with a reducing agent (DTT) to break disulfide bonds. Changes in Na^+^ current were assessed in response to oxidizing or reducing agents, whereas oocytes were maintained at a constant potential (−100 mV). The response to amiloride (10 μM) was measured to determine the ENaC-dependent component of the whole cell Na^+^ current. As mentioned previously, ENaCs not only conduct Na^+^ but are inhibited by extracellular Na^+^, a phenomenon referred to as Na^+^ self-inhibition (3, 14). This inhibitory response to Na^+^ is assessed by rapidly changing the solution bathing oocytes from a low (1 mM) to a high (110 mM) [Na^+^] while oocytes are maintained at a constant potential (−100 mV). The change in bath [Na^+^] was accompanied by a rapid increase in inward Na^+^ current, which reaches a peak current (Ipeak) and was followed by a slow decay in current to a steady-state current (Iss), reflecting Na^+^ self-inhibition. The percent of inhibition following Ipeak was calculated as described under the Experimental procedures section. Previous work has shown that the magnitude of the Na^+^ self-inhibition response correlates with ENaC open probability (42). In selected experiments, the Na^+^ self-inhibition response was examined before and after application of a reducing or oxidating reagent. In other experiments, only the later was examined.

### Channels with Cys substitutions of conserved acidic residues in the β11–β12 linker respond to sulfhydryl reagents in a subunit-dependent manner

**Figure 2:**
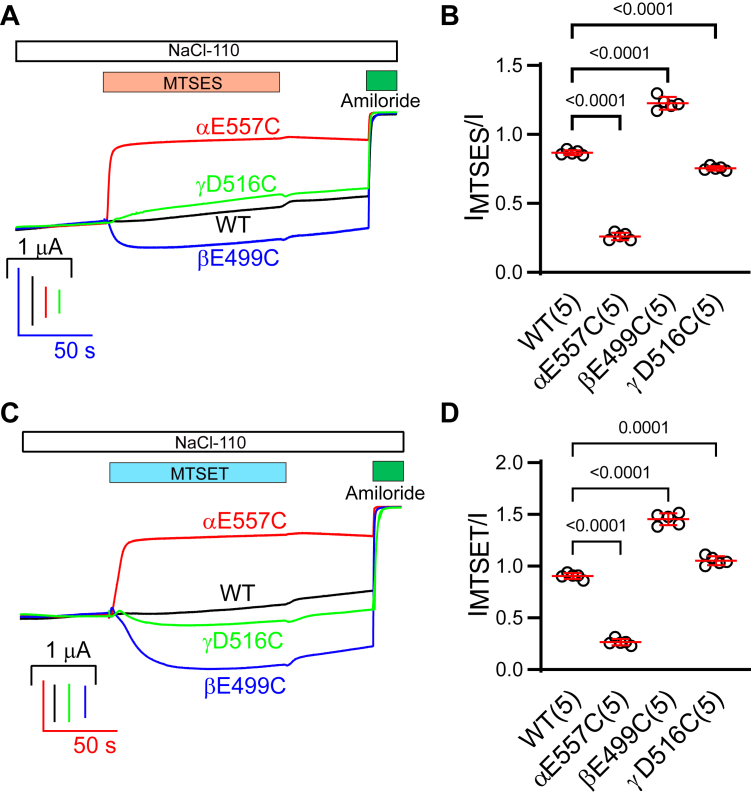
**Effects of MTS on Cys-substituted mutant ENaCs.***A* and *C*, representative traces showing the effect of MTSES or MTSET on αE557Cβγ (*red*), αβE499Cγ (*blue*), and αβγD516C (*green*) channels. Oocytes were clamped at −100 mV. After 2 mM MTSES perfusion (*orange bar*) or 1 mM MTSET perfusion (*blue bar*) and a 110 mM Na^+^ buffer (*white bar*) washout, 10 μM amiloride (*green bar*) was applied to determine the amiloride-sensitive current. Traces were superimposed by aligning the basal currents prior to MTSES or MTSET treatment and the currents after amiloride application. The time scale was the same, and the current scales were shown in *vertical lines*. *B* and *D*, scatter plots of I_MTSES_/I or I_MTSET_/I ratios presenting the responses of WT and mutant channels to the MTS reagents. I and I_MTSES_ or I_MTSET_ are amiloride-sensitive current before and after MTSES or MTSET perfusion. Bars are mean ± SD. Numbers in the parentheses are numbers of oocytes used in the experiment. One-way ANOVA and Dunnett post hoc test were conducted for variance analysis. ENaC, epithelial Na^+^ channel; MTSES, sodium (2-sulfonatoethyl) methanethiosulfonate; MTSET, [2-(trimethylammonium) ethyl] methanethiosulfonate bromide.

To further explore the functional roles of the β11–β12 linker acidic residues, we examined the effects of sulfhydryl reagents on the activity of ENaCs with Cys substitutions at αE557, βE499, or γD516 (Fig. 1). As shown in Figure 2, sodium (2-sulfonatoethyl) methanethiosulfonate (MTSES) significantly reduced or increased the currents in oocytes expressing αE557Cβγ or αβE499Cγ, respectively, when compared with wildtype channels. It also moderately reduced αβγD516C channel activity (Fig. 2, *A* and *B*). Similarly, [2-(trimethylammonium) ethyl] methanethiosulfonate bromide (MTSET) significantly reduced αE557Cβγ activity and increased αβE499Cγ and αβγD516C activity (Fig. 2, *C* and *D*). These results suggest these β11–β12 acidic residues have roles in modulating ENaC activity, in agreement with prior studies (38, 39, 40).

### Crosslinking αN510C and βE499C at the α/β subunit interface activates ENaC

**Figure 3:**
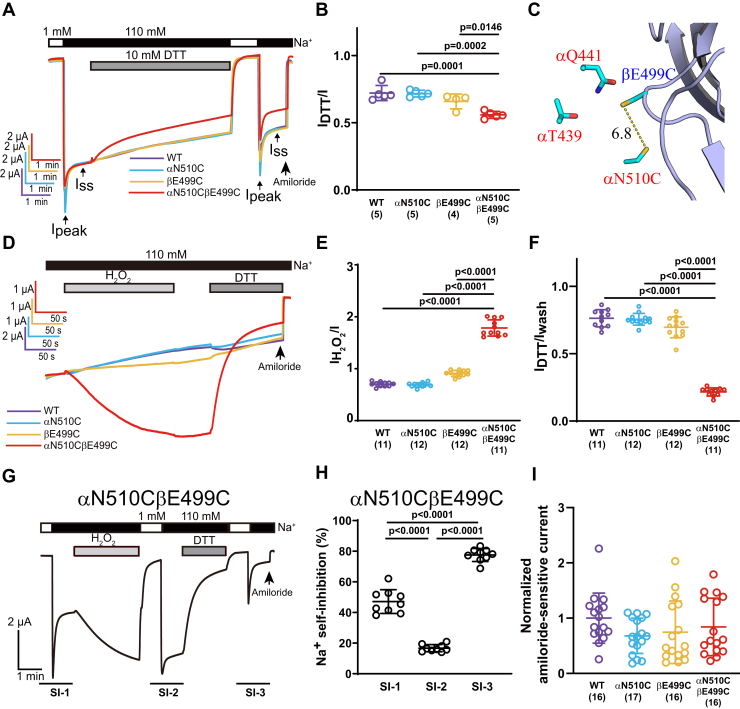
**Crosslinking palm domain αN510C and βE499C activated ENaC.***A*, representative recordings from oocytes expressing WT (*purple*), αN510Cβγ (*blue*), αβE499Cγ (*orange*), and αN510CβE499Cγ (*red*) ENaCs showing the effects of DTT and Na^+^ self-inhibition responses before and after DTT. Na^+^ self-inhibition was examined by switching Na^+^ bath solution from 1 mM (*white bar*) to 110 mM (*black bar*). Ipeak and Iss represent the peak current after switching from low to high [Na^+^], and the steady-state current measured 40 s after Ipeak, respectively. 10 mM DTT (*dark gray bar*) was applied for 5 min. Amiloride (10 μM) was added to the bath as indicated by an *arrow*. Traces were superimposed by aligning the currents prior to DTT application. *B*, scatter plot of I_DTT_/I. I and I_DTT_ were amiloride-sensitive current before and after DTT. Dot plots of WT, αN510Cβγ, αβE499Cγ, and αN510CβE499Cγ are presented in colors matching their traces. Bars are mean ± SD. Numbers in the parentheses are numbers of oocytes used in the experiment. *C*, a mouse ENaC model showing the relative position of βE499C and αN510C. Side chains of βE499C and αN510C were modeled by PyMol using the default rotamers. The distance was measured from sulfur to sulfur on the two Cys residues. Carbon, oxygen, nitrogen, and sulfur are shown in *cyan*, *red*, *blue*, and *yellow*, respectively. *D*, current traces from oocytes expressing WT, αN510Cβγ, αβE499Cγ, and αN510CβE499Cγ ENaCs are shown in colors consistent with *A*. H_2_O_2_ (0.045%, *light gray bar*) was applied in 110 mM Na^+^ bath solution for 3 min and washed out for 1 min before adding 10 mM DTT (*dark gray bar*) for 2 min and amiloride (10 μM, *arrow*). *E*, dot plots of I_H2O2_/I. I and I_H2O2_ were currents measured before and after 0.045% H_2_O_2_, respectively. *F*, dot plots of I_DTT_/Iwash. Iwash and I_DTT_ were measured immediately prior to and at the end of DTT application. Bars are mean ± SD. *G*, representative trace presenting the change of Na^+^ self-inhibition responses before and after 0.045% H_2_O_2_ (3 min) and after 10 mM DTT (2 min) in an oocyte expressing αN510CβE499Cγ. *H*, dot plots of Na^+^ self-inhibition (%) measured prior to H_2_O_2_ (self-inhibition (SI)-1), after H_2_O_2_ (SI-2) and after DTT (SI-3) in *G*. Values were calculated using the formula: 100 × (Ipeak-Iss)/Ipeak. The *p* values were obtained using repeated-measures ANOVA and Tukey's post hoc test. *I*, normalized amiloride-sensitive currents represent basal channel activity prior to either DTT or H_2_O_2_ treatment. Data were from experiments as shown in *A* and *D*. Amiloride-sensitive currents from all oocytes of the same batch were normalized to the mean of the currents in oocytes expressing wildtype ENaCs. The amiloride-sensitive currents from the three batches of oocytes expressing wildtype channels were 1.9 ± 1.5 μA (n = 5), 5.6 ± 1.5 μA (n = 6), and 6.1 ± 1.8 μA (n = 5). Since data from two groups did not pass normality test, Kruskal–Wallis nonparametric test was used for statistical analysis. There was no significant difference among the four groups (*p* > 0.05). The *p* values in *B*, *E*, and *F* were from one-way ANOVA and Tukey's post hoc tests. ENaC, epithelial Na^+^ channel.

To explore the functional interactions between αN510C in the α-subunit palm domain β10 strand and βE499C in the β-subunit β11–β12 linker (Fig. 3*C*), channels with 0, 1 or 2 Cys-substituted subunits were coexpressed with complementary wildtype subunits in *Xenopus* oocytes. Whole-cell currents in oocytes expressing wildtype or mutant ENaCs were measured at −100 mV (membrane potential). After assessing the Na^+^ self-inhibition response, 10 mM DTT was added to the bath solution. DTT did not significantly alter currents in oocytes expressing wildtype ENaC (Fig. 3*A*), consistent with previous observations (43). The slow decrease in current over time likely reflects the well-known rundown in ENaC activity under a continuous clamping at hyperpolarizing potential (44). Interestingly, in contrast to the continuous rundown in WT and single Cys mutants, DTT treatment caused a modest but rapid inhibition of the double Cys mutant (αN510CβE499Cγ, Fig. 3*A*). The current in the presence of DTT relative to basal current (I_DTT_/I) was significantly lower than that observed with wildtype ENaC or channels with a single Cys substitution (Fig. 3*B*). These results suggest that DTT inhibited αN510CβE499Cγ channels by breaking a disulfide bond spontaneously formed between the two Cys residues.

The aforementioned results suggested that a disulfide bond between αN510C and βE499C activates ENaC. Nevertheless, the DTT effect on αN510CβE499Cγ channel was modest, raising the possibility that only some channels formed disulfide bridges between αN510C and βE499C. We suspected that an oxidizing reagent would activate αN510CβE499Cγ channels by promoting the disulfide bridging and would enhance the subsequent response to DTT. To enhance disulfide bond crosslinking of αN510C and βE499C, we exposed ENaC-expressing oocytes to hydrogen peroxide (H_2_O_2_; 0.045%) prior to DTT (10 mM) treatment. Modest decreases in whole-cell currents were observed in oocytes expressing either wildtype, αN510Cβγ, or αβE499Cγ channels in response to 0.045% H_2_O_2_ (Fig. 3, *D* and *E*). In contrast, oocytes expressing αN510CβE499Cγ channels responded to H_2_O_2_ with a large and significant increase in current (I_H2O2_/I = 1.78 ± 0.16, n = 11, *p* < 0.0001 *versus* 0.70 ± 0.04, n = 11 for WT; *p* < 0.0001 *versus* 0.69 ± 0.04, n = 12 for αN510Cβγ; *p* < 0.0001 *versus* 0.91 ± 0.06, n = 12 for αβE499Cγ), which was sustained following H_2_O_2_ washout (Fig. 3, *D* and *E*). Subsequent treatment with 10 mM DTT led to a large fall in current only in oocytes expressing αN510CβE499Cγ channels (Fig. 3, *D* and *F*). Moreover, the effect of DTT on αN510CβE499Cγ channels was greater than what would be expected from a simple reversal of the H_2_O_2_-induced current increase. This likely reflected an additive effect of DTT on both populations of channels with pre-existing and H_2_O_2_-induced disulfide bonds. These results suggest that crosslinking these two residues activated the mutant channels, whereas breaking the bond inhibited the channels.

We predicted that the activated channels would exhibit a reduced Na^+^ self-inhibition response. This is what we observed. The Na^+^ self-inhibition response of αN510CβE499Cγ channels was significantly blunted after treatment with H_2_O_2_. Following DTT treatment, the Na^+^ self-inhibition response was more robust than the response under baseline conditions (Fig. 3, *G* and *H*). Again, the later likely reflected the effect of DTT on H_2_O_2_-oxidized channels as well as naturally bridged channels. Basal channel currents prior to either DTT or H_2_O_2_ did not significantly differ in oocytes expressing wildtype or mutant channels (*p* > 0.05, Fig. 3*I*).

### Crosslinking αQ441C and βE499C at the α/β subunit interface favors low channel activity

**Figure 4:**
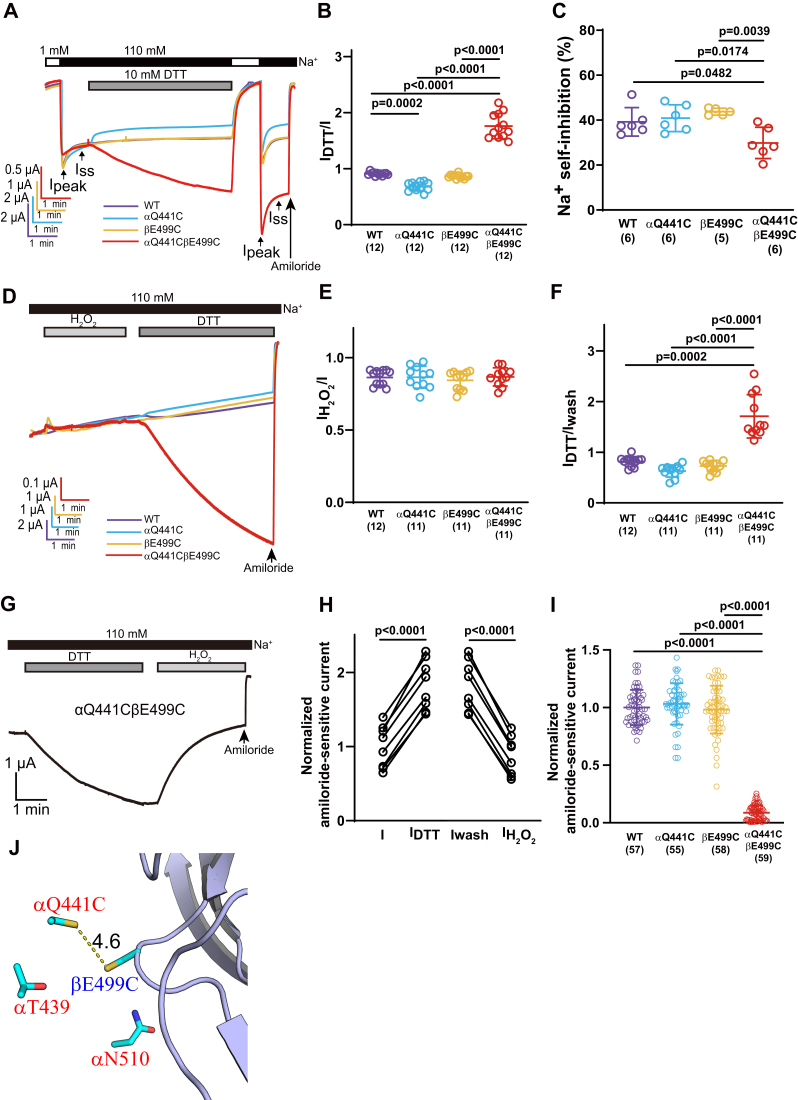
**Crosslinking thumb domain αQ441C and palm domain β11–β12 linker βE499C transitioned ENaC to a low-activity state with a blunted Na**^**+**^**self-inhibition response.***A*, representative recording showing the effect of 10 mM DTT on WT, αQ441Cβγ, αβE499Cγ, and αQ441CβE499Cγ channels. Current traces of WT (*purple*), αQ441Cβγ (*blue*), αβE499Cγ (*orange*), and αQ441CβE499Cγ (*red*) are superimposed by aligning the currents prior to DTT application. About 10 mM DTT was applied for 5 min. Ipeak and Iss represent the peak current after switching from low to high [Na^+^] and the steady-state current measured 40 s after Ipeak, respectively. *B*, dot plots of the I_DTT_/I for WT and mutant channels. The I_DTT_ and I were the currents measured 5 min after and immediately before DTT treatment, respectively. Data were from the experiments shown in *A*. WT, αQ441Cβγ, αβE499Cγ, and αQ441CβE499Cγ are presented in color consistent with their traces. *C*, dot plots of Na^+^ self-inhibition (%) of WT and mutant channels shown in colors matching traces in *A*. *D*, representative current recordings showing the effect of 0.045% H_2_O_2_ and 10 mM DTT on WT, αQ441Cβγ, αβE499Cγ, and αQ441CβE499Cγ channels. H_2_O_2_ was applied for 3 min, followed by 0.5 min wash out and then 5 min DTT treatment. *E*, dot plots of I_H2O2_/I. The I_H2O2_ and I were the currents measured after and immediately before H_2_O_2_ treatment, respectively. *F*, dot plots of I_DTT_/Iwash. The I_DTT_ and Iwash were measured after and immediately before DTT application (*i.e.*, after washout of H_2_O_2_), respectively. *G*, current trace showing the current changes responding to DTT (4 min), DTT washout (0.5 min), and H_2_O_2_ (3 min) in the same oocyte expressing αQ441CβE499Cγ. *H*, normalized amiloride-sensitive currents (n = 8) were the amiloride-sensitive currents measured immediately before DTT (I), after DTT (I_DTT_), after DTT washout (Iwash), and after H_2_O_2_ (I_H2O2_) that were divided by the mean of the current immediately before DTT. *p* Values were calculated by repeated-measures ANOVA and Tukey's post hoc test. In *B*, *C*, *E*, and *F*, bars are mean ± SD, with numbers of oocytes shown in the parentheses, and one-way ANOVA and Tukey's post hoc test were used to obtain *p* values. *I*, normalized amiloride-sensitive currents were amiloride-sensitive currents divided by mean of the amiloride-sensitive current in the same batch of oocytes expressing wildtype ENaC. Data were from two batches of oocytes in which the amiloride-sensitive currents in wildtype-expressing oocytes were 5.9 ± 0.6 μA (n = 27) and 4.7 ± 0.9 μA (n = 30). The *p* values were from Kruskal–Wallis nonparametric test with Dunn’s multiple comparisons test as data from three groups did not pass a normality test. The experiment was performed to compare expressed currents of the wildtype and mutant channels, and the data were not from experiments shown in *A* and *D*. *J*, mouse ENaC model showing the relative locations of βE499C and its adjacent αENaC residues. ENaC, epithelial Na^+^ channel.

We examined whether other residues with Cys substitutions in the vicinity of βE499C formed functional crosslinks. αQ441 is in the thumb domain α4 helix and in proximity to βE499 in the β11–β12 linker (Fig. 4*J*). Oocytes expressing αQ441CβE499Cγ channels, responded to the reducing agent DTT with a significant increase in current, which was not seen in oocytes expressing wildtype or channels with a single Cys substitution (I_DTT_/I = 1.76 ± 0.23, n = 12, *p* < 0. 0001 *versus* 0.91 ± 0.03, n = 12 for WT; *p* < 0.0001 *versus* I_DTT_/I = 0.68 ± 0.08, n = 12 for αQ441Cβγ; *p* < 0.0001 *versus* I_DTT_/I = 0.86 ± 0.04, n = 12 for αβE499Cγ; Fig. 4, *A* and *B*). A small inhibition of current in response to DTT was observed in αQ441Cβγ channels (I_DTT_/I = 0.68 ± 0.08, n = 12, *p* = 0.0002 for αQ441Cβγ *versus* I_DTT_/I = 0.91 ± 0.03, n = 12 for WT, Fig. 4, *A* and *B*) for an unknown reason. The large effect of DTT specifically in the double Cys mutant channels was consistent with a disulfide bridge that occurred naturally in the absence of an oxidizing reagent.

Treatment of wildtype or mutant channels with the oxidizing agent H_2_O_2_ was associated with minimal changes in currents (Fig. 4, *D* and *E*), whereas subsequent treatment with DTT led to a selective increase in current of αQ441CβE499Cγ channels (Fig. 4, *D* and *F*). When cells expressing αQ441CβE499Cγ channels were pretreated with DTT, subsequent treatment with H_2_O_2_ led to a large decrease in current (Fig. 4, *G* and *H*). These results suggest that a disulfide bond was spontaneously formed between αQ441C and βE499C in the absence of an added oxidant, and the bridging rendered the mutant channels in a low-activity state. To demonstrate that αQ441CβE499Cγ channels indeed have low ENaC activity, we expressed wildtype, αQ441Cβγ, αβE499Cγ, and αQ441CβE499Cγ channels in oocytes and measured amiloride-sensitive currents. As shown in Figure 4*I*, oocytes expressing αQ441CβE499Cγ channels had significantly lower currents than oocytes expressing wildtype and single mutant channels.

The Na^+^ self-inhibition response of αQ441CβE499Cγ channels (21 ± 11%, n = 12) was significantly less than that of wildtype (32 ± 9%, n = 12), αQ441Cβγ (32 ± 11%, n = 12), and αβE499Cγ (34 ± 9%, n = 12, Fig. 4, *A* and *C*). For αQ441CβE499Cγ channels, DTT not only activated the channel but also significantly increased the Na^+^ self-inhibition magnitude of αQ441CβE499Cγ (36 ± 12%, n = 12, *p* < 0.0001 *versus* the first response prior to DTT (see aforementioned) from paired Student's *t* test. These results are in contrast to the inverse relationship between the magnitude of Na^+^ self-inhibition and ENaC activity that has been frequently observed in mutagenesis studies (see Discussion section) (41, 45, 46).

### Introduced Cys at other sites adjacent to αQ441C, αN510C, and βE499C at the α/β subunit interface do not respond to reducing or oxidizing reagent

**Figure 5:**
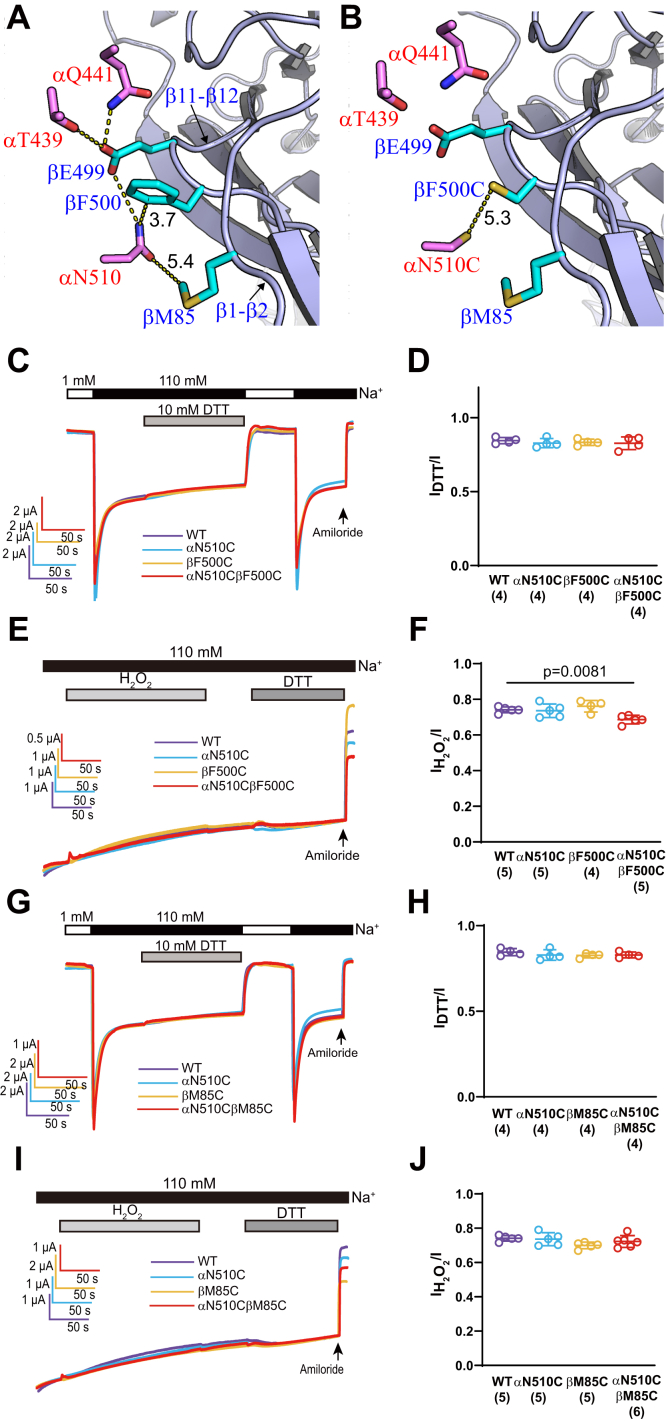
**αN510CβF500Cγ and αN510CβM85Cγ channels did not respond to DTT and H**_**2**_**O**_**2**_**.***A*, a mouse ENaC model showing selected residues at the α/β subunit interface. Portion of βENaC is shown in *blue*. Only three αENaC side chains are shown for clarity. Carbons in βENaC side chains are shown in *cyan*, and carbons in αENaC side chains are shown in *purple*. Distances are the minimal distances measured between nonhydrogen side-chain atoms of the two residues. *B*, a mutant model showing βF500C and αN510C pair. *C*–*F*, current traces and summary data to probe if αN510C and βF500C could be crosslinked. *G*–*J*, current traces and summary data to probe if αN510C and βM85C could be crosslinked. *C*–*J*, presented as for Figures 3 and 4. In *C* and *G*, DTT was applied for 2 min. In *E* and *I*, H_2_O_2_ was applied for 3 min, after wash out for 1 min, DTT was applied for 2 min. The *p* value was from one-way ANOVA and Tukey’s post hoc analysis. H_2_O_2_, hydrogen peroxide.

**Figure 6:**
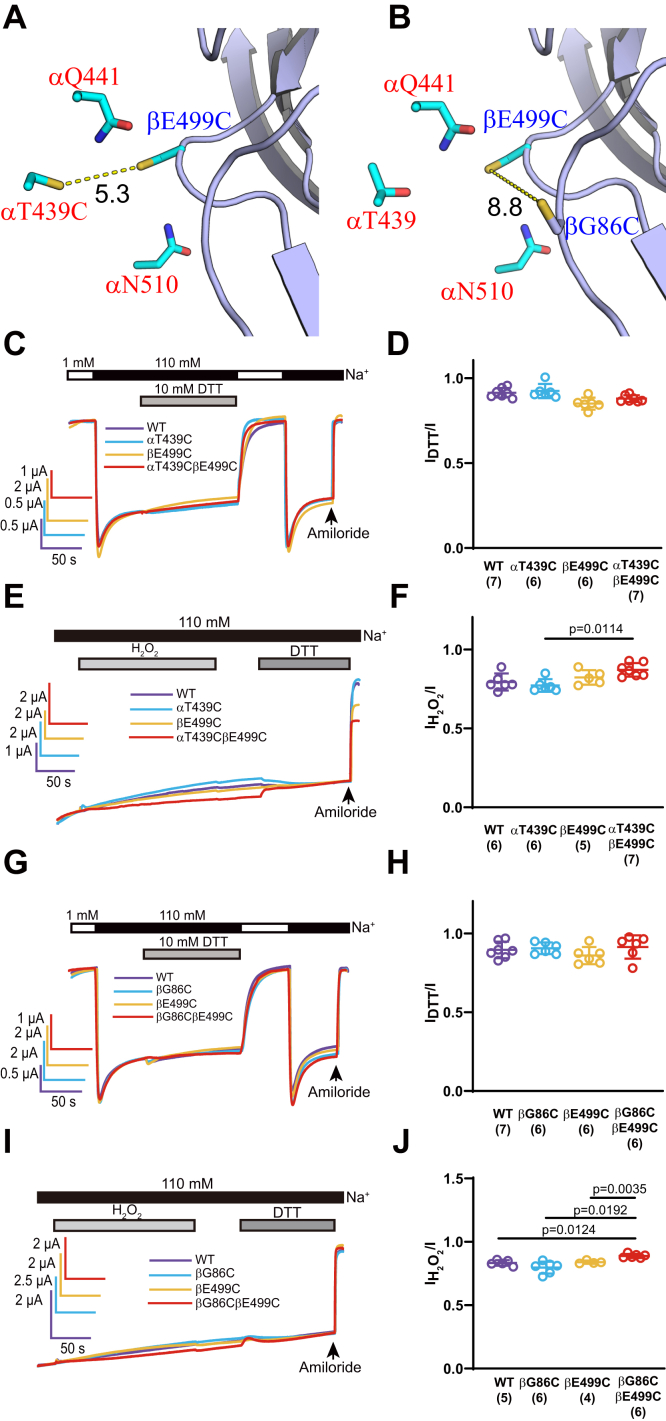
**αT439CβE499Cγ and αβG86CE499Cγ channels did not respond to DTT and H**_**2**_**O**_**2**_**.***A*, a mouse ENaC model, similar to Figure 4*J*, highlighting βE499C and αT439C, the target of crosslinking in this figure. *B*, a similar model to *A*, showing the relative locations of βE499C and βG86C. *C*–*F*, current traces and summary data to probe if βE499C and αT439C could be crosslinked. *G*–*J*, current traces and summary data to probe if βE499C and βM85C could be crosslinked. All *C*–*J* panels are presented as for Figures 3 and 4. In *C* and *G*, DTT was applied for 2 min. In *E* and *I*, H_2_O_2_ was applied for 3 min, after wash out for 1 min, DTT was applied for 2 min. The *p* values were from one-way ANOVA and Tukey’s post hoc analysis. ENaC, epithelial Na^+^ channel; H_2_O_2_, hydrogen peroxide.

We examined the effects of reducing and oxidizing reagents on channels with introduced Cys residues at other adjacent sites at the α/β subunit interface, including αN510CβF500Cγ, αN510CβM85Cγ, and αT439CβE499Cγ (Figs. 5, *A* and *B* and 6A). Significant changes of current were not observed following treatments with either H_2_O_2_ or DTT (Figs. 5 and 6), highlighting the specificity of the functional crosslinking we observed between αN510, αQ441, and βE499. We also assessed if a pair of Cys residues within β subunit (βE499C and βG86C) could form spontaneous or induced disulfide bond (Fig. 6*B*). A lack of effect of either DTT or H_2_O_2_ (Fig. 6, *G*–*J*) on αβG86C–E499Cγ channels suggested this was not the case. A similar pair of Cys residues within β11–β12 and β1–β2 linkers in ASIC1a were reported to form a disulfide bond, leading to dramatically slowed desensitization (32).

### Crosslinking αE557C and γQ398C at the α/γ subunit interface favors a low-activity state

**Figure 7:**
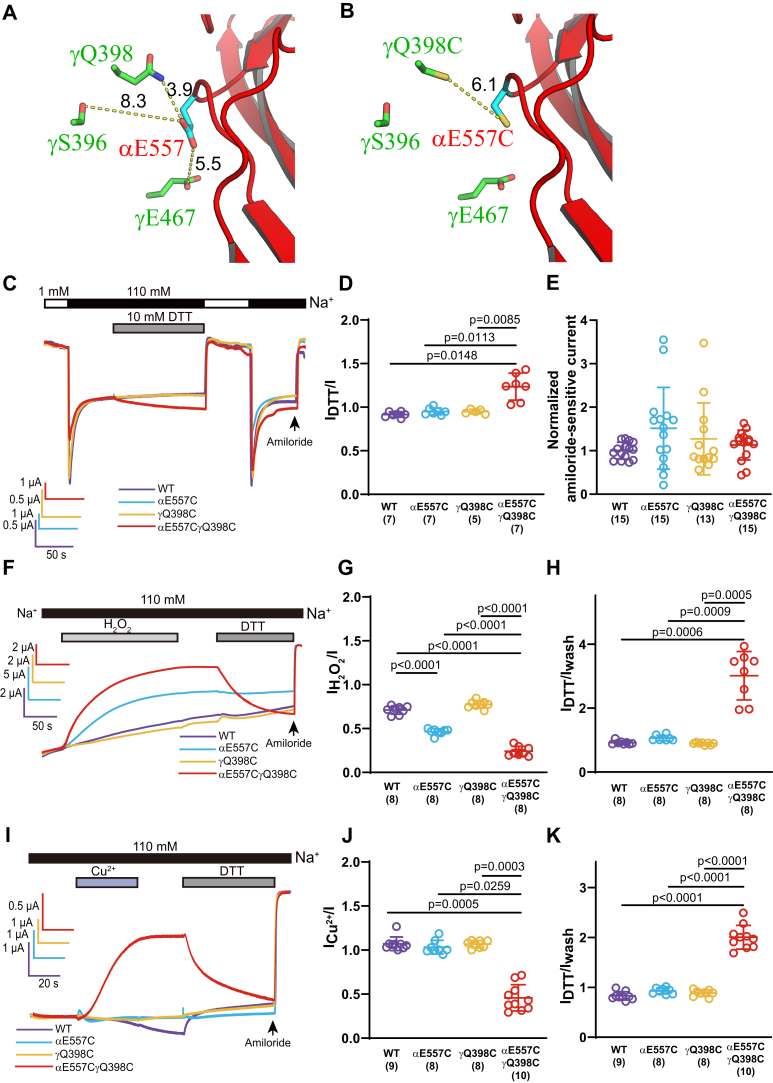
**Crosslinking αE557C and γQ398C favors low channel activity.***A*, a mouse ENaC model showing αE557 (side chain carbon in *cyan*) of β11–β12 linker and its adjacent γ subunit residues (side chain carbon in *green*) at the α/γ subunit interface. The shortest distances (Å) between two residues, measured using PyMol, are shown as *yellow dotted lines*. *B*, a similar model to *A* showing the relative position and distance of αE557C and γQ398C. *C*, representative recordings showing the effect of 10 mM DTT (2 min) on WT (*purple*), αE557Cβγ (*blue*), αβγQ398C (*orange*), and αE557CβγQ398C (*red*) channels. *D*, dot plots of I_DTT_/I from experiments shown in *C*. *E*, normalized amiloride-sensitive currents representing basal channel activity prior to either DTT or H_2_O_2_ treatment as shown in *C* and *F*. Amiloride-sensitive currents from all oocytes of the same batch were normalized to the mean current in oocytes of the same batch expressing wildtype ENaC. The amiloride-sensitive currents from the four batches of oocytes expressing wildtype channels were 4.1 ± 0.9 μA (n = 3), 1.2 ± 0.2 μA (n = 4), 2.6 ± 0.6 μA (n = 3), and 8.2 ± 1.7 μA (n = 5). Since data from one group did not pass normality test, Kruskal–Wallis nonparametric test was used for statistical analysis. There was no significant difference among the four groups (*p* > 0.05). *F*, representative recordings showing the effect of 0.045% H_2_O_2_ on WT, αE557Cβγ, αβγQ398C, and αE557CβγQ398C channels. H_2_O_2_ was applied for 3 min, after wash out for 1 min, DTT was applied for 2 min. *G* and *H*, dot plots presenting I_H2O2_/I and I_DTT_/Iwash that show the effect of H_2_O_2_ and the subsequent effect of DTT on WT and mutant channels. *I*, representative recordings showing the effects of 1 μM Cu^2+^ on WT, αE557Cβγ, αβγQ398C, and αE557CβγQ398C channels. Oocytes were perfused with 1 μM Cu^2+^ (*blue gray bar*) for 40 s to induce a disulfide crosslink, followed by 10 mM DTT for 1 min. *J* and *K*, dot plots of I_Cu2+_/I and I_DTT_/Iwash. Amiloride-sensitive currents were measured before (I) and after Cu^2+^ (I_Cu2+_), after Cu^2+^ washout (Iwash), and after DTT (I_DTT_). Bars are mean ± SD. Numbers in the parentheses are numbers of oocytes used in the experiment. *p* Values were calculated *via* one-way ANOVA and Tukey’s post hoc test. ENaC, epithelial Na^+^ channel; H_2_O_2_, hydrogen peroxide.

We next examined whether disulfide bonds between introduced Cys residues at the α/γ subunit interface altered ENaC function. In the γ subunit, γQ398 is homologous to αQ441 (Figs. 7*A* and 1C). In the α subunit, αE557 is homologous to βE499 (Figs. 7*A* and 1C). The relative locations of these residues are shown in Figure 7, *A* and *B*. We observed that DTT treatment led to a modest but significant activation of αE557CβγQ398C channels (I_DTT_/I = 1.23 ± 0.15, n = 7, *p* = 0.015 *versus* 0.92 ± 0.03, n = 7 for WT; *p* = 0.011 *versus* 0.95 ± 0.04, n = 7 for αE557Cβγ; *p* = 0.009 *versus* 0.95 ± 0.03), n = 5 for αβγQ398C; Fig. 7, *C* and *D*). Furthermore, αE557CβγQ398C channels responded to H_2_O_2_ with a large and significant reduction in current (I_H2O2_/I = 0.24 ± 0.06, n = 8, *p* < 0.0001 *versus* 0.71 ± 0.05, n = 8 for WT; *p* < 0.0001 *versus* 0.46 ± 0.03, n = 8 for αE557Cβγ; *p* < 0.0001 *versus* 0.78 ± 0.04, n = 8 for αβγQ398C; Fig. 7, *F* and *G*). The H_2_O_2_-induced reduction in αE557CβγQ398C currents was reversed following treatment with DTT (Fig. 7, *F* and *H*). αE557Cβγ channels also responded to H_2_O_2_ with a significant reduction in current (I _H2O2_/I = 0.46 ± 0.03, n = 8, *p* < 0.0001 *versus* 0.71 ± 0.05, n = 8 for WT). However, the H_2_O_2_-induced inhibition was not reversed by DTT (Fig. 7, *F*–*H*). Basal channel activity in oocytes expressing wildtype or mutant channels was similar prior to either DTT or H_2_O_2_ (*p* > 0.05, Fig. 7*E*).

To validate an induced disulfide bond between αE557C and γQ398C and further explore interactions between the two residues, we took advantage of the fact that two introduced Cys residues in close proximity can coordinate a metal ion, forming a metal-mediated crosslink (47). We examined whether Cu^2+^ could bridge αE557C and γQ398C. Treatment of oocytes expressing αE557CβγQ398C channels with 1 μM Cu^2+^ led to a rapid and large reduction in current that persisted after removing Cu^2+^ from the bath solution. In contrast, Cu^2+^ did not reduce currents in oocytes expressing WT, αE557Cβγ, or αβγQ398C channels (αE557CβγQ398C I_Cu2+_/I = 0.46 ± 0.15, n = 10, *p* = 0.0005 *versus* 1.07 ± 0.08, n = 9 for WT; *p* = 0.026 *versus* 1.03 ± 0.08, n = 8 for αE557Cβγ; *p* = 0. 0003 *versus* 1.07 ± 0.04, n = 8 for αβγQ398C, Fig. 7, *I* and *J*). Subsequent treatment with 10 mM DTT reversed the Cu^2+^-induced inhibition of αE557CβγQ398C channels, whereas little or no change in current was observed in oocytes expressing WT, αE557Cβγ, or αβγQ398C channels (Fig. 7, *I* and *K*). These results suggest that crosslinking αE557C and γQ398C at the α/γ interface maintains the channel in a low-activity state. Interestingly, αE557C and γQ398C are homologous to the βE499C and αQ441C within the β/α interface, where crosslinking also kept the channel in a low-activity state (Fig. 4).

## Discussion

**Figure 8:**
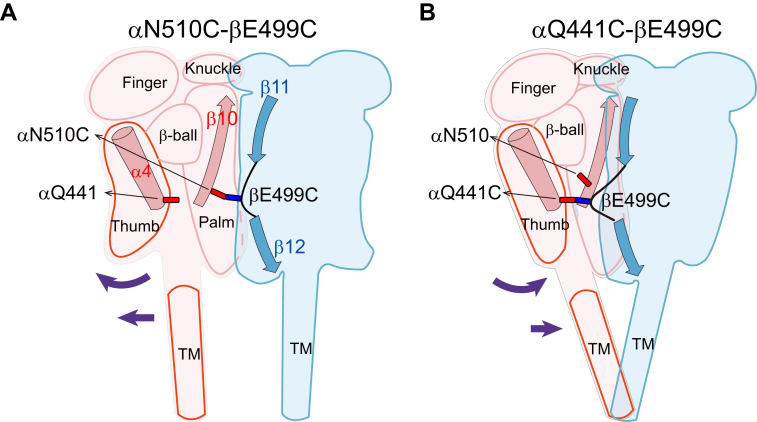
**Proposed mechanisms for the opposite effect of crosslinking βE499C to αN510C and αQ441C.***A*, an illustration showing a proposed mechanism underlying ENaC activation by crosslinking βE499C and αN510C. For clarity, only α and β ENaC subunits are shown in *light pink* and *light blue*, respectively. Individual extracellular domains of α subunit are depicted. The β10 strand and α4 helix of αENaC, together with β11 strand, β11–β12 linker, and β12 strand of βENaC are also shown. Side chains of the α and β residues are displayed as *sticks*. *Purple arrows* indicate potential movement of transmembrane (TM) domains and the lower thumb in response to βE499C and αN510C crosslinking. We propose that crosslinking βE499C and αN510C strengths interaction between the two palm domain residues and weakens the interaction between βE499 and thumb domain αQ441. As a result, the α-subunit lower thumb domain swings away from the α/β interface, favoring an open state TM conformation. *B*, a model showing a possible mechanism behind ENaC inhibition by crosslinking βE499C and αQ441C. We propose that crosslinking palm domain βE499C and thumb domain αQ441C strengths the interaction between the two residues and enhances contacts between the α thumb domain and β palm domain. This crosslink swings the α subunit lower thumb toward the α/β interface, favoring a closed TM conformation. ENaC, epithelial Na^+^ channel.

We introduced Cys residues at specific sites at ENaC subunit interfaces to explore the structural transitions during ENaC gating. Using the oxidizing agent H_2_O_2_ or metal ion Cu^2+^ to facilitate Cys crosslinks and the reducing agent DTT to release disulfide bonds, we examined the functional effects of intersubunit interactions at selected sites. Our results indicate that crosslinking αN510C and βE449C activated ENaC. In contrast, crosslinking αQ441C and βE499C, αE557C and γQ398C inhibited ENaC. We propose that residues, at or in the vicinity of these sites, facilitate interactions across subunit interfaces that help stabilize the channel in an open state or closed state. In essence, these residues constitute microswitches that can be triggered to transmit conformational changes that occur during ENaC gating transitions (Fig. 8). While we have not directly assessed channel gating, the changes in current that we are examining, in all likelihood, reflect changes in channel gating. It is difficult to explain the rapid and often reversible changes we are seeing on changes in ENaC trafficking and number of channels at the cell surface, and we are not perturbing the transmembrane regions where we might observe changes in single channel conductance.

Inducing an α/β-subunit crosslink between αN510C in palm domain β10 strand and βE499C in the palm domain β11–β12 linker promoted channel activation, whereas crosslinking αQ441C in the α4 helix of thumb domain and βE499C led to channel inhibition. This shift in bridging partners of βE499C (with αN510C or αQ441C) had a dramatic effect on the conformational states of ENaC, suggesting that intersubunit interactions involving βE499 and neighboring residues modulate channel gating. We propose that βE499, αN510, and αQ441 form a microswitch at α/β subunit interface that help stabilize ENaC in an open state or closed state, respectively. This switch can be triggered during intrinsic gating and in response to extracellular Na^+^ and other factors that impact ENaC gating behavior. A function role of the β11–β12 linker βE499 in regulating ENaC gating is also supported by the observed activation of αβE499Cγ channel by MTS reagents (Fig. 2).

The exact structural changes that occur in response to a Cys crosslink (or loss of a crosslink) that maintain channels in a specific (high or low) activity state are not known and will require high-resolution structures of ENaC in closed and open states to elucidate structural transitions associated with changes in ENaC gating that can then be confirmed by additional functional studies. As outlined in Figure 8, we hypothesize that crosslinking specific sites in the α-subunit palm domain β10 strand and the β-subunit palm domain β11–β12 linker (αN510C and βE499C) constrain these subunits at the mid palm domain level, altering interactions between α-subunit thumb domain and β-subunit palm domain and facilitating a transition in the transmembrane helices to an open conformation state. In contrast, crosslinking the α-subunit thumb domain α4 helix and β-subunit palm domain β11–β12 linker (αQ441C–βE499C) strengthen the interaction between these two domains, prompting a transmembrane helical conformation that stabilizes the channel in a closed state.

We found that the specific functional interactions at the α/β subunit interface were conserved at the α/γ subunit interface. Similar to an αQ441C and βE499C bridge, crosslinking αE557C in the palm domain β11–β12 linker and γQ398C in α4 helix of thumb domain trapped channels at a low-activity state. We speculate that β11–β12 linker αE557 and thumb domain γQ398 may form another microswitch at the α/γ subunit interface in ENaC gating. The functional changes in both pairs at α/β and α/γ interfaces are consistent with the notion that constraining thumb domain and palm domain at subunit interfaces favors a closed state (48, 49, 50). In ASIC1a, introduction of a disulfide bridge between thumb domain N357C of one subunit and palm domain β1–β2 linker T84C of another subunit blocked channel activation by protons, apparently favoring a closed state (32). These findings suggest that thumb and palm domain interactions at a subunit interface are a common feature in the regulated gating of ENaCs and ASICs.

In the assessment of crosslinking αE557C and γQ398C, H_2_O_2_ induced an unexpected inhibition on αE557C channels (Fig. 7, *F* and *G*). Given its insensitivity to DTT, the H_2_O_2_-induced inhibition might reflect oxidation of the thiol group (-SH) of αE557C to sulfinic acid (-S=O(OH)) or sulfonic acid (-S(=O)_2_(OH)). These oxidation reactions are not reversed by reducing agents (51). While it is not clear why αE557C is particularly sensitive to oxidation, it is known that susceptibility to oxidation of thiols is usually correlated to its acidity, which is influenced by the presence of polar residues or hydrogen bonds (52). As shown in Figure 7*A*, αE557 is in proximity to several polar residues including γS396, γQ398, and γE467. Interestingly, we observed a similar H_2_O_2_-induced inhibition in γE467C channels (L. Z. *et al.*, unpublished observation). The effects of H_2_O_2_ on both αE557C and γE467C preclude a clear assessment of a potential bridge between these Cys residues. Nevertheless, the surprising effect highlights the significant role of αE557 in regulating ENaC activity. The same may be true for γE467, as suggested in a recent report (41).

Members of the ENaC/degenerin family have evolved as ion channels with large extracellular domains with complex folds, where channel activity is highly regulated by extracellular factors. For ENaC, these factors include Na^+^ and other metals, H^+^, Cl^−^, proteases, and shear stress (3, 14). ASICs are regulated by extracellular H^+^ and specific small proteins that bind to and regulate channel activity (53). Degenerin channels in *Caenorhabditis elegans* are mechanoactivated channels (54, 55, 56). It is important to understand the extracellular structural transitions that occur in response to these channel activators or inhibitors and lead to transitions in the transmembrane helices that stabilize channels in a high- or low-activity state. Our results provide insights regarding possible structural transitions within the extracellular ENaC subunits that affect channel gating in response to extracellular factors. Furthermore, our observations in this study may advance an understanding of the allosteric regulations of ENaC-related channels.

Aside from transporting Na^+^, extracellular Na^+^ is one of the key regulators of ENaC through binding to an extracellular site in the α subunit based on functional as well as structural studies (15, 29). The inhibitory response of extracellular Na^+^, referred to as Na^+^ self-inhibition, transitions channels to a low-activity state. Open probability of ENaC is, in general, proportional to the magnitude of Na^+^ self-inhibition (3, 14, 42). Other extracellular regulators, including proteases, modulate ENaC activity in part by altering Na^+^ self-inhibition (3, 14, 15, 16, 26, 29, 37, 46, 49, 57, 58, 59, 60, 61, 62, 63, 64, 65, 66, 67, 68, 69, 70, 71, 72). We examined the Na^+^ self-inhibition response of the double Cys mutant channels before and after inducing or breaking a disulfide bond to determine if it was altered. Interestingly, we found that these functional crosslinks differentially affected the Na^+^ self-inhibition response. For αN510CβE499Cγ channels, crosslinking increased channel current with a blunted Na^+^ self-inhibition response, whereas releasing the disulfide bond reduced channel current and enhanced Na^+^ self-inhibition (Fig. 3, *G* and *H*). This relationship between channel activity and the magnitude of the Na^+^ self-inhibition response is strikingly similar to what has been observed in gain-of-function mutations and specific ENaC activators that affect channel gating (20, 21, 26, 37, 38, 39, 40, 42, 45, 46, 49, 62, 63, 64, 65, 68, 70, 73, 74, 75, 76, 77).

In contrast to αN510CβE499Cγ channels, crosslinked αQ441C–βE499C channels exhibited both reduced channel currents and a blunted Na^+^ self-inhibition (Fig. 4*C*). These channels were activated by DTT, along with a restored Na^+^ self-inhibition response. Our results suggest that αQ441CβE499Cγ channels are crosslinked under baseline conditions, and that this crosslink largely prevents conformational changes induced by extracellular Na^+^. This is reminiscent of the previously described αK477C–βV85C crosslinked channels (50). The Na^+^ self-inhibition response can be readily observed by monitoring current changes in response to a rapid change in the bath [Na^+^], reflecting a conformational change that impacts the open probability of ENaC. Our results suggest that crosslinked αQ441CβE499Cγ channels are trapped in a low-activity (*i.e.*, low open probability) state, even in the presence of a low (1 mM) bath [Na^+^]. Consistent with this notion, Collier *et al.* (50) reported a low open probability of crosslinked αK477CβV85C (also within the α/β subunit interface) in the presence of 1 mM extracellular Li^+^, along with a reduced Na^+^ self-inhibition response. DTT relieved this locked low-activity state, permitting conformational changes in response to bath [Na^+^] changes, similar to the response of wildtype ENaC.

In summary, we identified multiple pairs of extracellular domain residues where introduced Cys side chains across subunit interfaces formed either spontaneous or oxidant-induced disulfide bridges. These crosslinks resulted in significant changes in ENaC activity that were attributable to altered channel gating. Interestingly, certain crosslinking events disrupted Na^+^ self-inhibition response, whereas disulfide bridge formations at other sites retained the inhibitory response. We propose certain endogenous residues at or near these crosslinking sites interact across these interfaces forming key microswitches to facilitate structural changes during channel transitions between closed and open states. There are likely additional sites present in the extracellular domains that mediate domain–domain interactions. Identification and characterization of these interacting sites will greatly enhance our understanding of the mechanisms of ENaC gating and its regulation and should facilitate the discovery of specific therapeutic agents selectively targeting ENaC gating.

## Experimental procedures

### Chemicals

MTSES and MTSET were from Toronto Research Chemicals. All other chemicals were from Sigma–Aldrich unless otherwise stated.

### Site-directed mutagenesis in ENaC

Point mutations were introduced into mouse α, β, and γ ENaC plasmid DNAs using QuickChange Ⅱ XL site-directed mutagenesis kit (Agilent). The DNA sequences, including those of wildtype and mutant ENaC, were confirmed by direct sequencing at the Genomics Research Core of University of Pittsburgh. Wildtype and mutant RNAs were made from the linearized DNA using T3 RNA *in vitro* transcription kit and purified with RNA Cleanup Kit (Thermo Fisher Scientific Inc). RNA quality was verified by denaturing RNA gel analysis, and RNA concentrations were estimated by a spectrophotometer. One mutation (αN510C) was made previously (41).

### *Xenopus* oocyte isolation

Oocytes harvested from female *Xenopus laevis* were separated into small pieces and treated with 2 mg/ml type II collagenase plus 2 mg/ml trypsin inhibitor for 55 to 60 min. Dispersed oocytes were immersed in solution containing 100 mM K_2_HPO_4_ solution plus 1 mg/ml bovine serum albumin for 15 min before wash and incubation with modified Barth’s solution (88 mM NaCl, 1 mM KCl, 2.4 mM NaHCO_3_, 15 mM Hepes, 0.3 mM Ca(NO_3_)_2_, 0.41 mM CaCl_2_, 0.82 mM MgSO_4_, 10 μg/ml streptomycin sulfate, 100 μg/ml gentamycin sulfate, and 10 μg/ml sodium penicillin, pH 7.4). The frog surgery protocol was approved by the University of Pittsburgh’s Institutional Animal Care and Use Committee.

### Injection of oocytes and expression of ENaC

Oocytes were kept at 18 °C and injected at room temperature the day after isolation. Wildtype or mutant mouse ENaC α, β, and γ subunit RNAs were injected into stage Ⅴ or Ⅵ oocytes in a 1:1:1 manner. Either 0.5 ng, 1 ng, or 2 ng ENaC subunit RNAs were injected each time to achieve an appropriate current level for experiments. Injected oocytes were incubated at 18 °C in a modified Barth’s solution: 88 mM NaCl, 1 mM KCl, 2.4 mM NaHCO_3_, 15 mM Hepes, 0.3 mM Ca(NO_3_)_2_, 0.41 mM CaCl_2_, 0.82 mM MgSO_4_, 10 μg/ml streptomycin sulfate, 100 μg/ml gentamycin sulfate, and 10 μg/ml sodium penicillin, pH 7.4.

### Two-electrode voltage clamp

Whole cell Na^+^ currents were measured using a two-electrode voltage clamp at room temperature 1 or 2 days after injection. Oocytes were placed in a chamber and perfused with constant flow (NaCl-110: 110 mM NaCl, 2 mM KCl, 2 mM CaCl_2_, and 10 mM Hepes, pH 7.4) and clamped at −100 mV (membrane potential) using glass pipettes filled with 3 M KCl solution. Recorded signals were amplified and digitized using TEV200A Voltage Clamp Amplifier (Dagan Corporation), DigiData 1440A, and Clampex 10.4 software (Molecular Devices).

### Effects of MTSET and MTSES

MTSES and MTSET were freshly dissolved in regular bath solution (NaCl-110). MTSES (2 mM) solution was used for experiments within 2 h. MTSET (1 mM) solution was prepared immediately prior to use. MTSES and MTSET solutions were perfused for 2 min. Amiloride-sensitive Na^+^ currents prior to and after MTSES or MTSET application were measured as I and I_MTSES_ or I_MTSET_, respectively.

### Na^+^-self inhibition response

Na^+^-self inhibition was examined by perfusing oocytes with NaCl-1 bath solution (1 mM NaCl, 109 mM *N*-methyl-d-glucamine, 2 mM KCl, 2 mM CaCl_2_, and 10 mM Hepes, pH 7.4) for 60 s and rapidly switching to NaCl-110 bath solution described previously and perfusing for 60 s. During this process, current recorded increased rapidly to reach a peak value (Ipeak) and then gradually decreased to a steady-state level (Iss, measured at 40 s after Ipeak). Na^+^ self-inhibition magnitude was calculated as percentage of (Ipeak-Iss)/Ipeak.

### Crosslinking introduced Cys residues and reducing disulfide bonds

DTT (200 mM) stock solutions were prepared on the day of experiment in NaCl-110. DTT stock solutions were diluted with NaCl-110 to 10 mM preceding recording. H_2_O_2_ (3%) solutions were stored at room temperature and protected from light. H_2_O_2_ (0.045%) solutions were prepared by diluting 3% H_2_O_2_ with NaCl-110 before recording. A 40 mM CuSO_4_ stock solution was prepared in ethanol and diluted to 1 μM with NaCl-110 before use. A 10 mM DTT, 0.045% H_2_O_2_, or 1 μM Cu^2+^ solution was applied extracellularly after the current became stable. At the end of each recording, 10 μM amiloride was applied to determine the amiloride-sensitive component of the whole-cell current. Amiloride-sensitive current before and after the treatment was measured using Clampfit 10.4 (Molecular Devices) and presented as I and I_DTT_, I_H2O2_, and I_Cu_. The ratios I_DTT_/I, I_H2O2_/I, and I_Cu_/I were calculated to show the effects of these reagents.

### Statistical analyses

Data are presented as mean ± SD. Shapiro–Wilk test was performed for normality determination. Significance was examined by Student’s *t* test with Welch’s correction for comparing two groups, one-way ANOVA followed by Tukey’s post hoc test for comparing multiple groups. *p* < 0.05 was regarded statistically significant. *p* Values were shown unless less than 0.0001. All analyses were performed using Prism 7 or 9 (GraphPad Software, Inc).

## Data availability

All data generated or analyzed during this study are included in the article and supporting information.

## Conflict of interest

The authors declare that they have no conflicts of interest with the contents of this article.
